# Non-coding-regulatory regions of human brain genes delineated by bacterial artificial chromosome knock-in mice

**DOI:** 10.1186/1741-7007-11-106

**Published:** 2013-10-14

**Authors:** Jean-François Schmouth, Mauro Castellarin, Stéphanie Laprise, Kathleen G Banks, Russell J Bonaguro, Simone C McInerny, Lisa Borretta, Mahsa Amirabbasi, Andrea J Korecki, Elodie Portales-Casamar, Gary Wilson, Lisa Dreolini, Steven JM Jones, Wyeth W Wasserman, Daniel Goldowitz, Robert A Holt, Elizabeth M Simpson

**Affiliations:** 1 Centre for Molecular Medicine and Therapeutics at the Child and Family Research Institute, University of British Columbia, Vancouver, British Columbia V5Z 4H4, Canada; 2 Genetics Graduate Program, University of British Columbia, Vancouver, British Columbia V6T 1Z2, Canada; 3 Canada’s Michael Smith Genome Sciences Centre, British Columbia Cancer Agency, Vancouver, British Columbia V5Z 4S6, Canada; 4 Department of Molecular Biology and Biochemistry, Simon Fraser University, Burnaby, British Columbia V5A 1S6, Canada; 5 Department of Medical Genetics, University of British Columbia, Vancouver, British Columbia V6T 1Z3, Canada; 6 Department of Psychiatry, University of British Columbia, Vancouver, British Columbia V6T 2A1, Canada

**Keywords:** Humanized mouse models, Brain expression pattern, Eye expression pattern, Brain development, Reporter gene, Transgenic mice, *Hprt* locus, High-throughput, Bacterial artificial chromosome

## Abstract

**Background:**

The next big challenge in human genetics is understanding the 98% of the genome that comprises non-coding DNA. Hidden in this DNA are sequences critical for gene regulation, and new experimental strategies are needed to understand the functional role of gene-regulation sequences in health and disease. In this study, we build upon our HuGX ('high-throughput human genes on the X chromosome’) strategy to expand our understanding of human gene regulation *in vivo*.

**Results:**

In all, ten human genes known to express in therapeutically important brain regions were chosen for study. For eight of these genes, human bacterial artificial chromosome clones were identified, retrofitted with a reporter, knocked single-copy into the *Hprt* locus in mouse embryonic stem cells, and mouse strains derived. Five of these human genes expressed in mouse, and all expressed in the adult brain region for which they were chosen. This defined the boundaries of the genomic DNA sufficient for brain expression, and refined our knowledge regarding the complexity of gene regulation. We also characterized for the first time the expression of human *MAOA* and *NR2F2*, two genes for which the mouse homologs have been extensively studied in the central nervous system (CNS), and *AMOTL1* and *NOV*, for which roles in CNS have been unclear.

**Conclusions:**

We have demonstrated the use of the HuGX strategy to functionally delineate non-coding-regulatory regions of therapeutically important human brain genes. Our results also show that a careful investigation, using publicly available resources and bioinformatics, can lead to accurate predictions of gene expression.

## Background

Over the past few decades, geneticists have primarily focused their research on protein-coding DNA sequences, leading to the identification of essentially all genes, the understanding of the molecular function for many of them, as well as the implications of gene mutations in human diseases and disorders. The study of protein-coding DNA sequences remains important, but also focuses on only a small fraction of the human genome (2%). The next big challenge in the field of human genetics lies in understanding the role of the remaining 98% of the genome, which comprises non-coding DNA sequences critical for gene regulation, chromosome function, and generation of untranslated RNAs [1]. New experimental strategies are needed to understand the functional role of non-coding sequences in health and disease. Pioneer examples in this work include large-scale efforts from the Encyclopedia of DNA Elements (ENCODE) consortium [2] seeking to catalog regulatory elements in the human genome, and the Pleiades Promoter Project [3] identifying brain-specific regulatory elements using humanized mouse models [4,5]. The latter project aimed at refining our understanding of regulatory elements, as well as providing researchers with novel tools for directed gene expression in restricted brain regions [5]. These tools were designed to be amenable to gene therapy as they were MiniPromoters of less than 4 kb, made entirely from human DNA elements, and selected for expression in 30 brain regions and cell types of therapeutic interest [5,6]. However, the bioinformatic approaches used for MiniPromoter design resulted in a biased selection for genes with low regulatory complexity, having well-defined and conserved non-coding regions that were close to the transcription start site (TSS) [5].

An additional set of ten genes, which were judged to be important for brain expression and/or relevance to disease, were omitted from Pleiades MiniPromoter development because they either had regulatory regions that were too large, too numerous candidate regulatory regions, or multiple TSS. For these genes, the Pleiades Promoter Project designed MaxiPromoters as an alternative [6]. A MaxiPromoter consists of a bacterial artificial chromosome (BAC) that has a reporter gene sequence (*lacZ* or *EGFP*) inserted at the start codon. For a BAC to be ideally suited for MaxiPromoter design, it has to span the whole predicted gene sequence plus extensive flanking intergenic sequences, but cannot contain the predicted promoter region of an adjoining gene.

These MaxiPromoters were used to test the veracity of the predicted regulatory regions, as well as to define the boundaries of the genomic DNA that were sufficient for brain-specific expression, thus leading to a refinement of our knowledge regarding the complexity of gene regulation. The strategy used to generate the mice for expression study *in vivo* was built on our method for high-throughput single-copy site-specific generation of humanized mouse models; entitled HuGX ('high-throughput human genes on the X chromosome’) [7]. Characterization of expression from the MaxiPromoter reporter construct was performed in development at embryonic day 12.5 (E12.5), postnatal day 7 (P7), and adult brain and eyes. In this study, we characterize for the first time the expression of human *MAOA*, and *NR2F2*, two genes for which the mouse homologs have been extensively studied in central nervous system (CNS) development [8-14], and *AMOTL1* and *NOV*, for which roles in CNS have been unclear in either species.

*AMOTL1* (angiomotin-like 1), initially known as junction-enriched and -associated protein (JEAP), encodes a member of the motin protein family [15,16]. The gene was selected for being enriched for expression in the thalamus, a brain region implicated in the cognitive impairment of early stage Huntington’s disease (HD) [17]. *In vitro* and *in vivo* mouse studies have demonstrated that the Amotl1 protein localizes at 'tight’ junctions in cells [15]. Amotl1 regulates sprouting angiogenesis by affecting tip cell migration, and cell-cell adhesion *in vivo*[18]. Northern blot analysis demonstrated high levels of transcripts of the mouse *Amotl1* gene in the brain, heart, lung, skeletal muscle, kidney, and uterus [16]. These results differed from previously reported immunohistochemical analysis demonstrating absence of expression in the brain, heart, and kidney [15]. Discrepancy between the studies can be partly explained by the existence of different isoforms of the Amotl1 protein, highlighting the need for further characterization [18].

*MAOA* (monoamine oxidase A) is a gene encoding a membrane-bound mitochondrial flavoprotein that deaminates monoaminergic neurotransmitters [11,19]. The gene was selected for expression in the locus coeruleus (LC), a component of the neuroadrenergic system that has been linked to the etiology of depressive illness [20]. In mice, characterization of *Maoa* by *in situ* hybridization and immunohistochemistry during CNS development demonstrated expression in a variety of neurons, including noradrenergic and adrenergic neurons as well as dopaminergic cells in the substantia nigra [21]. *Maoa* is expressed in neurons populating the developing brainstem, amygdala, cranial sensory ganglia, and the raphe [21]. Transient expression in cholinergic motor nuclei in the hindbrain, and in non-aminergic neurons populating the thalamus, hippocampus, and claustrum has also been detected during development [21]. In adult rodent brain, *Maoa* transcription is detected in neurons populating the cerebral cortices, the hippocampal formation (HPF), and the cerebellar granule cell layer [22]. *Maoa* knockout models implicate this gene as a regulator of neurochemical pathways, leading to increased levels of serotonin (5-hydroxytryptamine (5-HT)), norepinephrine, dopamine, and noradrenaline neurotransmitters in adult brain [23,24]. This increased level of neurotransmitters leads to behavioral abnormalities including aggression, which can be rescued by a *Maoa* forebrain-specific transgenic mouse model [23-26]. The role of *Maoa* in regulating neurotransmitters also impacts development by affecting telencephalic neural progenitors and retinal ganglion cell projections [12,14].

*NOV* (nephroblastoma overexpressed gene), belongs to the *CCN (CYR61, CTGF* and *NOV*) family of secreted matrix-associated signaling regulators [27]. The gene was selected for being enriched in the amygdala and basolateral complex, which includes brain regions that have been implicated in affective processing and memory [28]. The NOV protein stimulates fibroblast proliferation via a tyrosine phosphorylation dependent pathway [29]. In mice, *Nov* characterization by *in situ* hybridization revealed that the gene transcripts are first observed at E10 in a subset of dermomyotomal cells of muscular origin along the entire embryonic rostrocaudal axis [27]. In the CNS, *Nov* transcripts are first detected at E11.5 in scattered cells of the olfactory epithelium, the developing cochlea and the trigeminal ganglia [27]. Transcription observed in the olfactory epithelium extends later to cells populating the olfactory lobe (E13.5) [27]. At E12.5 and onward, *Nov* transcription is detected in muscle cell types of diverse developing tissues including vertebral muscles, limb muscles (femur, and hind foot), aorta, and other major vessels, maxillary muscles, and extraocular muscles [27]. Additionally, at E12.5, *Nov* transcription is observed in developing motor neurons in the ventral horns of the spinal cords [27]. *In situ* hybridization transcript localization on human fetal tissues revealed similar results and suggest that *NOV* plays an important role in neuronal differentiation [30]. Furthermore, investigation on prenatal lead exposure has revealed a reduction in the expression level of *Nov* in rat offspring hippocampus, demonstrating a potential mechanism by which lead exposure affects learning and memory [31]. Overall, these results highlight the need for further investigation of the role of *NOV* in central nervous system.

*NR2F2* (nuclear receptor 2f2), also known as *COUP-TFII*/*ARP1*, is a gene encoding for a transcription factor belonging to the orphan receptors group. Like *NOV*, *NR2F2* was selected for being enriched in the amygdala and basolateral complex, which includes brain regions that have been implicated in affective processing and memory [28]. In mice, transcription of *Nr2f2* by *in situ* hybridization during CNS development is first observed at embryonic day 8.5 (E8.5), peaks at E14 to 15, and declines after birth [9]. *Nr2f2* expression in the developing telencephalon is restricted to the caudal lateral domains, with positive staining in the medial ganglionic eminence, and caudal ganglionic eminence (CGE) [9,10]. *Nr2f2* function in the CGE is essential for the migration of inhibitory interneurons during forebrain development [10]. These interneurons migrate from the CGE via the caudal migratory stream to populate the neocortex, hippocampus, and amygdala [32,33]. Recent studies demonstrated a role for *Nr2f2*, and its closest relative (*Nr2f1*) in regulating the temporal specification of neural stem/progenitor cells in the ventricular zone of the developing CNS [13]. In the adult CNS, *Nr2f2* is expressed in a subpopulation of calretinin-positive interneurons in the postnatal cortex, and a population of amacrine cells in the mouse adult retina [8,10]. Finally, *Nr2f2* participates in the development and proper function of multiple organs, including the inner ear, limbs, skeletal muscles, heart, and pancreas [34-37]. The expression of *Nr2f2* in heart and pancreatic development highlights the role of this gene in regulating angiogenesis, a property that in turn affects tumor growth, and metastasis in cancer [38].

## Results and discussion

### High-throughput construction of humanized mice to study gene expression

Gene choosing for this study was based in part on serial analysis of gene expression (SAGE) profiling of brain regions of therapeutic interest, as well as data mining of both the Allen Mouse Brain Atlas (ABA) [39] and Brain Gene Expression Map (BGEM) [6,40-42]. The ten chosen human genes all had enriched expression of their mouse homologs in brain regions of therapeutic interest (Table 1). The expression pattern of these human genes was then analyzed in this study using mouse models generated through the HuGX method, that is, carrying a single copy of a human BAC MaxiPromoter reporter construct docked at the *Hprt* locus [7]. This approach was used to validate the genomic DNA boundaries that were predicted to be sufficient for adult brain-specific expression of the selected human genes, and to document the expression pattern resulting from the human MaxiPromoter constructs at different developmental stages in mouse.

**Table 1:** Eight out of ten constructs successfully modified for expression-pattern characterization in mice

**Brain region(s)**	**Gene**	**Ple**^**a**^	**Parental BAC name**	**Construct size (bp)**	**Reporter**	**BAC name**	**Retrofitting status**
Thalamus	*AMOTL1*	5	RP11-936P10	211,681	*lacZ*	bEMS90	Successful
Brainstem, pons, medulla	*GLRA1*	91	RP11-602K10	188,541	N/A	bEMS89	Failed^b^
Hippocampus, dentate gyrus	*LCT*	126	RP11-406M16	156,570	*EGFP*	bEMS75	Successful
Locus coeruleus	*MAOA*	127	RP11-475M12	197,191	*lacZ*	bEMS84	Successful
Hippocampus, Ammon’s horn	*NEUROD6*	132	RP11-463M14	N/A	N/A	N/A	Failed^c^
Basal nucleus of Meynert	*NGFR*	133	RP11-158L10	175,851	*lacZ*	bEMS88	Successful
Amygdala, basolateral complex	*NOV*	134	RP11-840I14	202,342	*lacZ*	bEMS91	Successful
Neurogenic regions	*NR2E1*	142	RP11-144P8	138,165	*lacZ*	bEMS86	Successful
Amygdala, basolateral complex	*NR2F2*	143	RP11-134D15	213,112	*lacZ*	bEMS85	Successful
Subthalamic nucleus	*PITX2*	158	RP11-268I1	200,209	*lacZ*	bEMS87	Successful

^a^Pleiades (Ple) promoter design number.

^b^*GLRA1* failed retrofitting with *Hprt* homology arms.

^c^*NEUROD6* failed BAC isolation from the library.

BAC bacterial artificial chromosome, N/A not applicable.

Of the ten selected genes, eight BAC MaxiPromoters were successfully constructed that contained an *Hprt* complementation cassette and a reporter gene (*lacZ* or *EGFP*) (Table 1). One planned BAC construct, RP11-463 M14 (*NEUROD6*), was abandoned due to rearrangement in the parental BAC clone, resulting in a smaller DNA insert than expected. Thus, using our retrofitting approach a success rate of 89% (8 out of 9) was obtained, with the largest construct spanning >213 kb (Table 1). The only retrofitting failure was partly due to an irresolvable primer design issue for *GLRA1* (Table 1). While many technical challenges can occur when using recombineering technology in a high-throughput manner, the high success rate obtained in our approach demonstrates the overall efficiency of the technology.

The eight retrofitted MaxiPromoter constructs were electroporated into embryonic stem cells (ESCs), and positive recombinant clones were selected, and screened as described in materials and methods. On average, 35% (range 20 to 53) correctly targeted clones were recovered for each construct (Table 2). For each MaxiPromoter, correctly targeted ESCs were microinjected to generate chimeric animals from which germline transmission was obtained; 100% success rate (8/8). MaxiPromoter expression was evaluated in E12.5 embryos, P7 brains, and adult brains and eyes. Gross dissection and whole body staining for expression investigation of adults was also performed. Based on these studies, 63% (5/8) MaxiPromoter strains were positive for expression of the human gene in mouse tissues (Table 3). Of the three negative strains, the failure to detect expression from the *LCT* MaxiPromoter could be attributable to sensitivity issues regarding the *EGFP* reporter gene. This fluorescence reporter was subsequently excluded from our single-copy insertion work [5], and replaced with the *lacZ* reporter, which offers greater sensitivity when direct readout of the enzymatic product is used. All other MaxiPromoter constructs were retrofitted with *lacZ*. The two additional negative results, for the *NGFR* and *PITX2* strains*,* could be attributable to undetected construct mutations. However, almost all the deletions that were detected, during screening for correctly targeted ESC clones (Table 2), were large and eliminated multiple polymerase chain reaction (PCR) assays. Furthermore, both the *NGFR* and *PITX2* strains were examined using the same multiple PCR assays, which showed that the BAC constructs had not changed in the mice. Hence, these negative results were more likely due to missing human gene regulatory regions, and/or the human regulatory regions present in the BAC being non-functional in the mouse genome. In parallel to our work, the Gene Expression Nervous System Atlas (GENSAT) project [43] reported positive brain expression using mouse *Pitx2* and *Ngfr* in random-insertion BAC*-*mouse models (*Pitx2*, RP24-215O15; *Ngfr*, RP23-138M22) [44]. Sequence comparison, using relative coordinates between human and mouse, revealed that our human BAC constructs (*PITX2*, RP11-268I1; *NGFR*, RP11-158 L10) were shorter than the mouse BACs used by GENSAT. *PITX2* was shorter at the 5′ end by approximately 30 kb, and *NGFR* was shorter at the 3′ end by approximately 40 kb. Thus, our work suggests a testable hypothesis that important regulatory elements reside in these differential regions. Finally, we characterized in detail the expression patterns observed from four of the five positive humanized mouse strains generated in this project: *AMOTL1*-*lacZ*, *MAOA*-*lacZ*, *NOV*-*lacZ* and *NR2F2*-*lacZ*. The detailed analysis of the *NR2E1*-*lacZ* strain was presented in a previous publication [45].

**Table 2:** **Eight novel *Hprt *** **targeted embryonic stem cell lines successfully generated**

**Gene**	**Parental ESC**		**ESC clones**		**Final ESC line (mEMS)**
	**Line (mEMS)**	**Genotype of parental ESC line**	**Number isolated**	**Correctly targeted (%)**	
*AMOTL1*	1202.04	B6129F1-*Gt(ROSA)26Sor*^+/+^, *Hprt*^*b-m3*^/Y	8	50	4645
*LCT*	1204.31	B6129F1-*Gt(ROSA)26Sor*^*tm1Sor/+*^, *Hprt*^*b-m3*^/Y	31	48	3305
*MAOA*	1202.04	B6129F1-*Gt(ROSA)26Sor*^*+/+*^, *Hprt*^*b-m3*^/Y	15	53	4442
*NGFR*	1202.04	B6129F1-*Gt(ROSA)26Sor*^*+/+*^, *Hprt*^*b-m3*^/Y	20	20	4583
*NOV*	1202.04	B6129F1-*Gt(ROSA)26Sor*^*+/+*^, *Hprt*^*b-m3*^/Y	24	42	4521
*NR2E1*	1204	B6129F1-*Gt(ROSA)26Sor*^*tm1Sor/+*^, *Hprt*^*b-m3*^/Y	29^a^	21	4751
*NR2F2*	1292.02	B6129F1-*A*^*w-J*^*/A*^*w*^, *Gt(ROSA)26Sor*^*tm1Sor/+*^, *Hprt*^*b-m3*^/Y	25^a^	28	4990
*PITX2*	1202.04	B6129F1-*Gt(ROSA)26Sor*^*+/+*^, *Hprt*^*b-m3*^/Y	15	20	4496

^a^Obtained from multiple electroporations.

ESC embryonic stem cell.

mEMS (mammalian cell lines Elizabeth M. Simpson).

**Table 3:** Summary of expression pattern from reporter mouse strains

**Gene**	**Embryo E12.5**	**P7 brain**	**Adult brain**	**Eye**	**Other**	**MMRRC strain name**	**MMRRC stock no.**
*AMOTL1*	Positive	Positive	Positive	Positive	Positive	B6.129P2(Cg)-*Hprt*^*tm66(Ple5-lacZ)Ems*^/Mmjax	012354
*LCT*	Negative	Negative	Negative	N/D	N/D	N/A	N/A
*MAOA*	Positive	Positive	Positive	Positive	Positive	B6.129P2(Cg)-*Hprt*^*tm68(Ple127-lacZ)Ems*^/Mmjax	012583
*NGFR*	Negative	Negative	Negative	Negative	Negative	N/A	N/A
*NOV*	Positive	Positive	Positive	Negative	Positive	B6.129P2(Cg)-*Hprt*^*tm69(Ple134-lacZ)Ems*^/Mmjax	012584
*NR2E1*	Positive	Positive	Positive	Positive	N/D	B6.129P2(Cg)-*Hprt*^*tm73(Ple142-lacZ)Ems*^/Mmjax	032962
*NR2F2*	Positive	Positive	Positive	Negative	Positive	B6.129P2(Cg)-*Hprt*^*tm75(Ple143-lacZ)Ems*^/Mmjax	014536
*PITX2*	Negative	Negative	Negative	Negative	Negative	N/A	N/A

MMRRC Mutant Mouse Regional Resource Center, N/A not applicable, N/D not determined.

### Human genes can be conditionally deleted using flanking functional *loxP* sites

**Figure 1:**
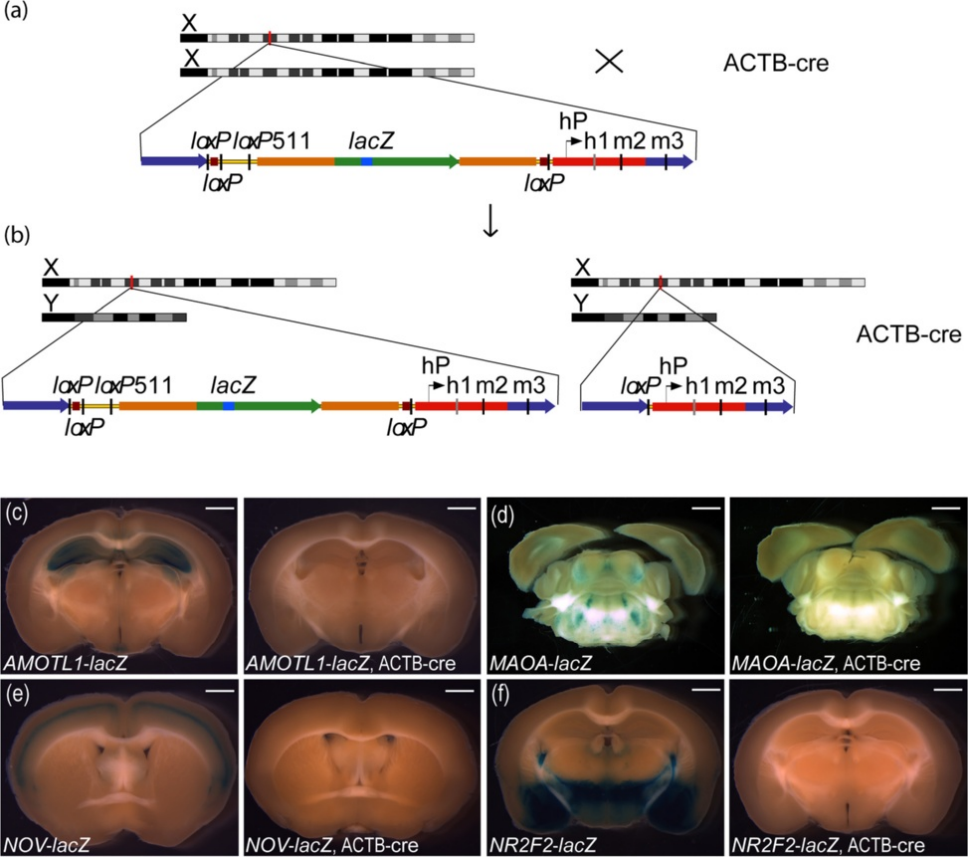
**Human bacterial artificial chromosomes can be targeted at *Hprt *by homologous recombination and, if desired, conditionally removed using cre recombinase. (a)** Integration into the mouse genome of the bacterial artificial chromosome (BAC)-*lacZ*-reporter constructs by homologous recombination results in the human gene in either direction relative to the X chromosome; this schematic presents one possible orientation. Regardless of orientation, each insertion resulted in the presence of four *loxP* sites in the genome (two wild-type and one 511 mutant at one end and one wild-type at the other end of the BAC insert). **(b)** Crossing the BAC-*lacZ-*reporter females to ACTB-cre males should result in the generation of two different male offspring; BAC-*lacZ*-reporter animals, wild-type for the ACTB-cre transgene; and BAC-*lacZ-*reporter animals carrying the ACTB-cre transgene. Only the reporter animals that are positive for the ACTB-cre gene should recombine the outer most *loxP* sites, resulting in excision of the BAC construct from the genome and leaving one *loxP* site. This would result in an absence of *lacZ-*positive signal. hP*,* human *HPRT* promoter; h1, human first exon; m2 and m3, mouse second and third exons; mouse homology arms (dark blue); *Hprt* coding regions (red); vector backbone (yellow with black edges); *Sac*B gene from BAC vector backbone (brown); 5′ and 3′ untranslated regions of the human gene (orange); coding region of the human gene (green); *lacZ* reporter gene (light blue). Schematic, not to scale. **(c-f)***lacZ* expression results from *AMOTL1*-*lacZ*, *MAOA*-*lacZ*, *NOV*-*lacZ*, and *NR2F2*-*lacZ* females bred to the ACTB-cre males are presented. *lacZ*-positive staining (blue) was detected in *AMOTL1*-*lacZ*, *MAOA*-*lacZ*, *NOV*-*lacZ*, and *NR2F2*-*lacZ* males not carrying the ACTB-cre allele whereas absence of staining was detected in males positive for ACTB-cre by genotyping (*AMOTL1*-*lacZ*, ACTB-cre; *MAOA*-*lacZ*, ACTB-cre; *NOV*-*lacZ*, ACTB-cre; *NR2F2*-*lacZ*, ACTB-cre), suggesting whole BAC excision from the genome. Scale bar: **(c-f)** 1 mm. N = 3 animals for all genotypes.

The HuGX method used in this study consisted of retrofitting existing human BAC constructs to carry an *Hprt* complementation cassette that allowed site specific targeting into the genome of *Hprt*^*b-m3*^ ESCs by homologous recombination [7]. The resulting complementation targeting event was detectable using described PCR assays [46]. The insertion of the *Hprt* complementation cassette by recombineering resulted in the disruption of a *Sac*B gene present on the original BAC vector, as well as the addition of *loxP* sites flanking the entire human gene. To verify that these *loxP* sites were functional in the final MaxiPromoter carrying strains, we bred *AMOTL1-lacZ*, *MAOA*-*lacZ*, *NOV*-*lacZ* and *NR2F2*-*lacZ* female mice to ACTB-cre male mice (Figure 1a,b), and examined the resulting brain expression patterns from male offspring. Positive animals for the complementation at the *Hprt* locus by genotyping were evaluated based on the presence or absence of the ACTB-cre transgene. The results showed that in all strains, *lacZ* staining (blue) was only detected in males, positive for the complementation at the *Hprt* locus and negative for the presence of the ACTB-cre transgene (*AMOTL1*-*lacZ*, *MAOA*-*lacZ*, *NOV*-*lacZ*, and *NR2F2*-*lacZ*) (first panel of Figure 1c-f). Absence of *lacZ* staining was only found in brains of the animals that were positive for both the complementation at the *Hprt* locus and the ACTB-cre transgene (*AMOTL1*-*lacZ*, ACTB-cre; *MAOA*-*lacZ*, ACTB-cre; *NOV*-*lacZ*, ACTB-cre; *NR2F2*-*lacZ*, ACTB-cre), suggesting complete excision of the BAC construct from the genome and proper function of the *loxP* sites (second panel of Figure 1c-f).

### Human *AMOTL1-lacZ* revealed staining in mature thalamic neurons in adult brain, and amacrine as well as ganglion cells in adult retina

**Figure 2:**
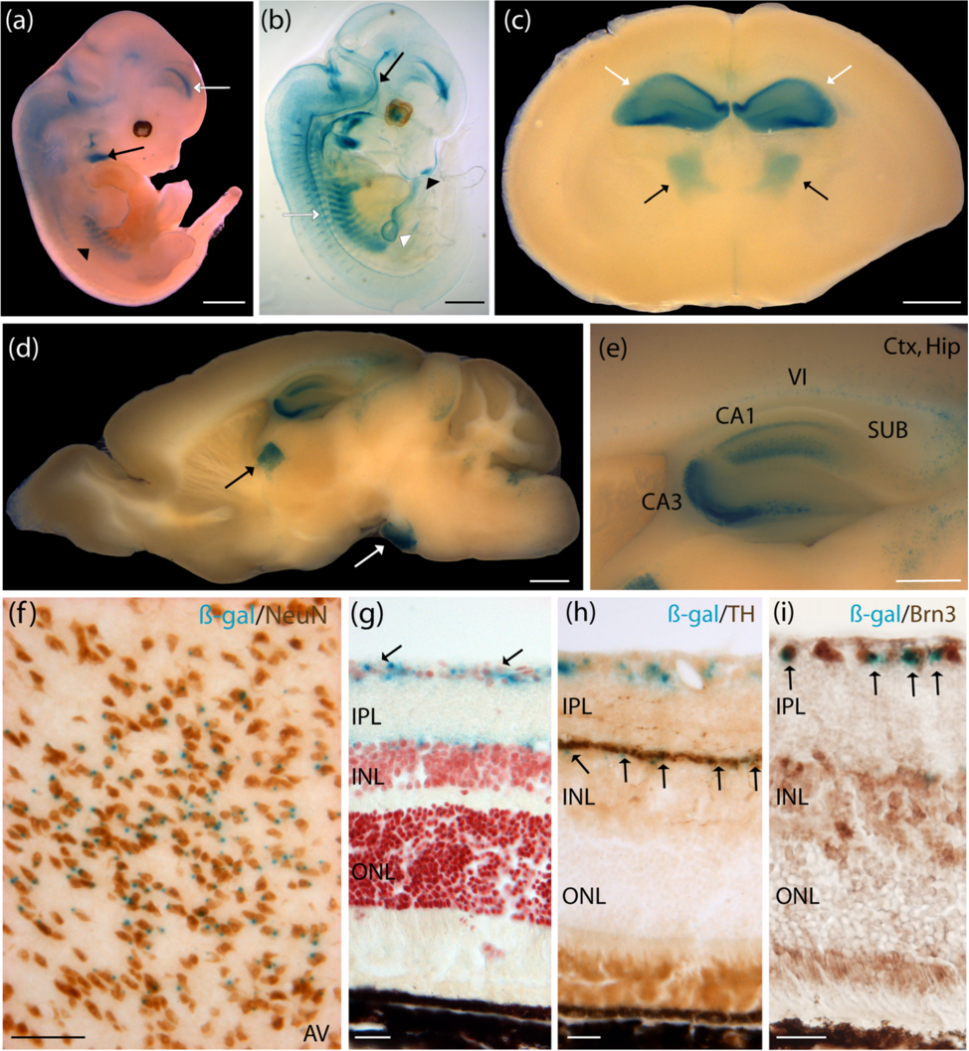
**Human *AMOTL1*-*lacZ *expressed in mature neurons in the thalamus in adult brain, amacrine and ganglion cells in adult retina, and major components of the vasculature system during development.** Expression analysis of the human *AMOTL1*-*lacZ* strain was examined using β-galactosidase (β-gal) histochemistry staining (blue). **(a)** E12.5 whole embryos stained in the jugular vein (black arrow), posterior cerebral artery (white arrow), and vertebral arteries (black arrowhead). **(b)** E12.5 cleared embryos additionally demonstrated staining in the basilary artery (black arrow), the dorsal aorta (white arrow), the omphalomesenteric vascular system (white arrowhead), and the lateral nasal prominence (black arrowhead). **(c)** P7 brains stained in the developing anterior thalamic nuclei (black arrows), and hippocampal formation (HPF) (white arrows). **(d)** Adult brains stained in the anteromedial and anteroventral thalamic nuclei (black arrow), and the pontine nuclei (white arrow). **(e)** The HPF showed sharp staining in the cornu ammonis fields (CA1, and CA3). Staining was also present in the dorsal subiculum and extended to the lower cortical layers with pronounce staining in layer VI. **(f)** Colocalization experiment using β-gal staining and neuronal nuclei (NeuN) immunohistochemistry (brown) performed on adult brain cryosections demonstrated expression of *AMOTL1*-*lacZ* in thalamic neurons. **(g)** Staining was detected in two layers of the adult retina, the inner limiting membrane (ILM) (black arrows), and the junction between the inner plexiform layer (IPL) and the ILM. **(h)** Colocalization experiment using β-gal staining and an anti-tyrosine hydroxylase (anti-TH) antibody (brown) performed on adult eye cryosections indicated colocalization of *AMOTL1*-*lacZ* in amacrine cells populating the inner nuclear layer (INL) (black arrows). **(i)** Colocalization experiment using β-gal staining and an anti-Brn3 antibody (brown) performed on adult eye cryosections indicated colocalization of *AMOTL1*-*lacZ* in ganglion cells populating the ganglion cell layer (GCL) (black arrows). AV, anteroventral thalamic nucleus; ctx, cortex; Hip, hippocampus; ONL, outer nuclear layer; SUB, subiculum. Scale bar: **(a**-**d)** 1 mm; **(e)** 500 μm; **(f)** 100 μm; **(g-i)** 20 μm.

*AMOTL1* expression was predicted to be in the thalamus in the adult brain. Whole mount *lacZ* E12.5 stained embryos revealed expression of the *AMOTL1* reporter construct in components of the vascular system such as the jugular vein, the posterior cerebral artery, and the vertebrate arteries (Figure 2a). Additional analyses using embryos subjected to a clearing protocol confirmed staining in the previously described regions, and revealed expression in the basilary artery, the dorsal aorta, and vertebrate arteries (Figure 2b). Staining was observed in the omphalomesenteric vascular system, and the lateral nasal prominence (Figure 2b). P7 developing mouse brain sections revealed expression in the anterior thalamic nuclei, and HPF (Figure 2c). Adult mouse brain sections revealed expression in the anteromedial, and anteroventral thalamic nuclei, the pontine nuclei, and the HPF, with sharp staining in the cornu ammonis fields (CA1 and CA3) (Figure 2d,e). Additional staining was detected in the subiculum and extended to the lower cortical layers with pronounced staining in layer VI (Figure 2e). On cryosections positive colocalization between the β-gal staining product (blue) and neuronal nuclei (NeuN)-specific antibody (brown) indicated that the *AMOTL1* reporter construct was expressed in mature neurons in all brain regions, including the anteroventral thalamic nuclei (Figure 2f). The β-gal-positive cells (blue) did not colocalize with glial fibrillary acidic protein (GFAP) (brown), a marker of astrocytes (data not shown). Further *AMOTL1-lacZ* expression pattern characterization revealed staining in the adult retina, extending from the inner limiting membrane (ILM) to the junction between the inner plexiform layer (IPL) and the inner nuclear layer (INL) (Figure 2g). The β-gal staining product (blue) detected at the junction of the IPL, and INL, colocalized with anti-tyrosine hydroxylase (anti-TH) (brown), demonstrating expression of the *AMOTL1* MaxiPromoter reporter construct in a subpopulation of amacrine cells found in the INL (Figure 2h) [47]. Colocalization using an anti-Brn3 antibody revealed expression in the ILM corresponding to ganglion cells in the retina (Figure 2i). Additional staining was found in the nasal structures, extending from the naris, to the nasopharynx, and at the sternum junction, in the extremities of the ribs in the *AMOTL1*-*lacZ* animals in gross dissections (data not shown).

### Human *MAOA-lacZ* revealed staining in TH-positive neurons in the locus coeruleus in adult brain as well as horizontal, and ganglion cells in adult retina

**Figure 3:**
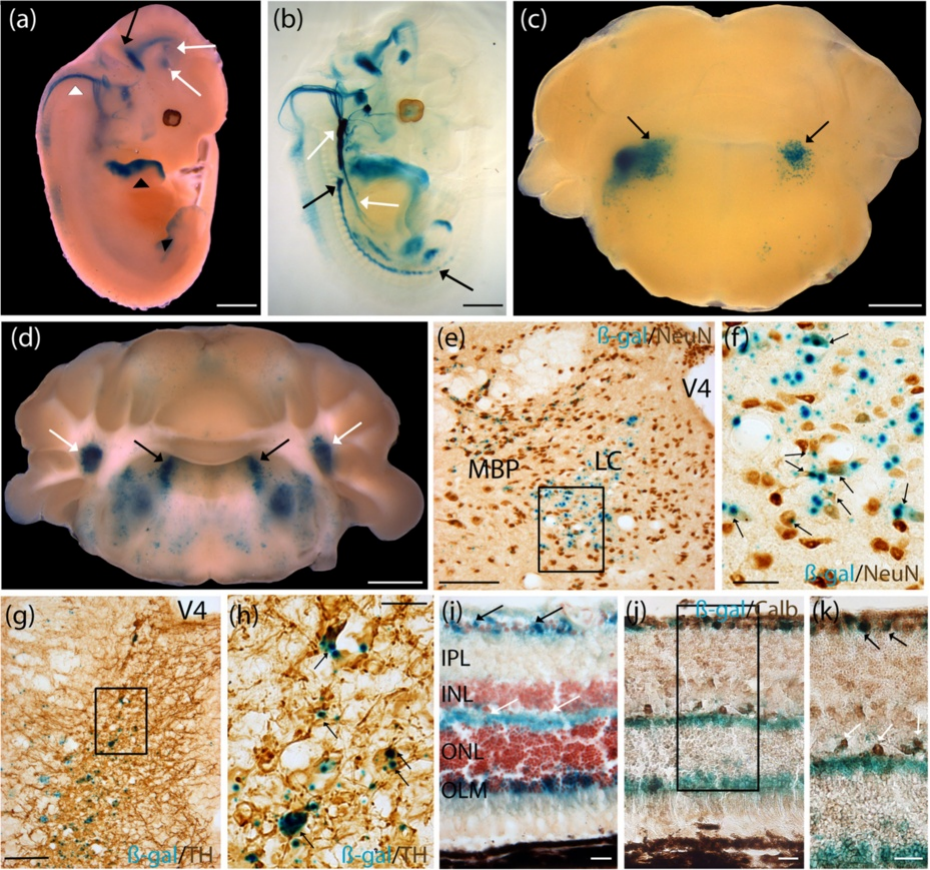
**Human *MAOA*-*lacZ *expressed in tyrosine hydroxylase (TH)-positive neurons in the locus coeruleus in adult brain, horizontal and ganglion cells in the adult retina, and several regions of the developing hindbrain.** Expression analysis of the human *MAOA*-*lacZ* strain was examined using β-galactosidase (β-gal) histochemistry (blue). **(a)** E12.5 whole embryos stained in the prepontine hindbrain (black arrow) that extended to the basal midbrain and the prosomeres 1 and 2 of the diencephalon (white arrows). Staining extended from the pontine hindbrain (pons proper) to the medullary hindbrain (medulla) (white arrowhead). Staining was notable in the anterior part of the developing limbs (black arrowhead). **(b)** E12.5 cleared embryos additionally demonstrated staining in fibers of the prepontine hindbrain, and the pontine hindbrain. Staining was present in fibers of the medullary hindbrain that extended into the thoracic cavity; and fibers of the ventral region of the somites that started in the upper thoracic cavity, and extended towards the posterior limbs. **(c)** P7 brains showed staining in nuclei surrounding the fourth ventricle, a region where the locus coeruleus (LC) is located (black arrows). **(d)** Adult brains stained in the LC (black arrows), and lateral cerebellar nuclei (white arrows). Staining was also present in the medial vestibular nuclei, and lateral vestibular nuclei. **(e)** Colocalization experiment using β-gal staining and neuronal nuclei (NeuN) immunohistochemistry (brown) performed on adult brain cryosections suggested expression of *MAOA*-*lacZ* in mature neurons in the LC. Sparse staining was detected in neurons populating the medial parabrachial nucleus. Boxed region in **(e)** is shown in **(f)**. **(f)** Higher magnification revealed expression of β-gal in neurons in the LC (black arrows). **(g)** Colocalization experiment using β-gal staining and an anti-TH immunohistochemistry (brown) performed on adult brain cryosections suggested expression of *MAOA*-*lacZ* in TH-positive neurons populating the LC. Boxed region in **(g)** is shown in **(h)**. **(h)** Higher magnification revealed expression of β-gal in TH-positive neurons in the LC (black arrows). **(i)** Staining was detected in the retina, extending from the inner limiting membrane (black arrows) to the outer plexiform layer (white arrows), and the outer limiting membrane (OLM). **(j**,**k)** Colocalization experiment using β-gal staining and an anti-calbindin (anti-Calb) immunohistochemistry (brown) performed on adult eye cryosections suggested expression of *MAOA*-*lacZ* in horizontal cells populating the OPL (white arrows), and ganglion cells populating the ganglion cell layer (black arrows). Boxed region in **(j)** is shown magnified in **(k)**. INL, inner nuclear layer; IPL, inner plexiform layer; MBP, medial parabrachial nucleus; ONL, outer nuclear layer; V4, fourth ventricle. Scale bar: **(a**-**d)** 1 mm; **(e**,**g)** 100 μm; **(f**,**h)** 25 μm; **(i**-**k)** 20 μm.

*MAOA* expression was predicted to be observed in the LC in the adult brain. Whole mount *lacZ* E12.5 stained embryos revealed expression in the prepontine hindbrain that extended to the basal midbrain and the prosomeres 1 and 2 of the diencephalon (Figure 3a). Staining extended from the pontine hindbrain (pons proper) to the medullary hindbrain (medulla) (Figure 3a). E12.5 cleared embryos confirmed staining in the previously described regions, and revealed expression of the *MAOA* reporter construct in major neuron fibers extending from the prepontine hindbrain, and the pontine hindbrain (Figure 3b). Staining was present in fibers of the medullary hindbrain that extended into the thoracic cavity; and fibers of the ventral region of the somites that started in the upper thoracic cavity, and extended towards the posterior limbs (Figure 3b). P7 developing mouse brain sections revealed expression at the lateral extremities of the fourth ventricle, demonstrating expression of the *MAOA* MaxiPromoter reporter construct in the region of the LC at this developmental stage (Figure 3c). Adult mouse-brain-section staining revealed high levels of expression in the LC and lateral cerebellar nuclei, as well as the medial and lateral vestibular nuclei (Figure 3d). Further colocalization experiments on cryosections revealed that the *MAOA*-*lacZ* reporter gene (blue) was expressed in mature neurons, positive for NeuN (brown), in all brain regions, including the LC and medial parabrachial nucleus (Figure 3e; higher magnification on the LC, Figure 3f). The β-gal staining product (blue) found in the LC colocalized with TH-positive cells (brown), revealing expression of the *MAOA* reporter construct in TH-positive neurons populating the LC (Figure 3g,h). The β-gal-positive cells (blue) in the LC did not colocalize with GFAP (brown), a marker of astrocytes (data not shown). Furthermore, *MAOA-lacZ* expression pattern characterization revealed staining in several layers in the adult retina such as the ILM, the outer plexiform layer (OPL), and the outer limiting membrane (Figure 3i). Calbindin is a marker for three different cell types in the retina: the ganglion cells that populate the ganglion cell layer (GCL), the amacrine cells found in the INL, and the horizontal cells populating the OPL [47]. Colocalization using an anti-Calbindin antibody revealed that the *MAOA*-*lacZ* construct was expressed in both the horizontal cells populating the OPL and the ganglion cells found in the GCL (Figure 3j,k). Additional staining was detected along the spinal cord in the *MAOA*-*lacZ* animals in gross dissections (data not shown).

### Human *NOV-lacZ* revealed staining in neurons populating the hippocampal formation, basolateral amygdaloid nuclei, and cortical layers in adult brain

**Figure 4:**
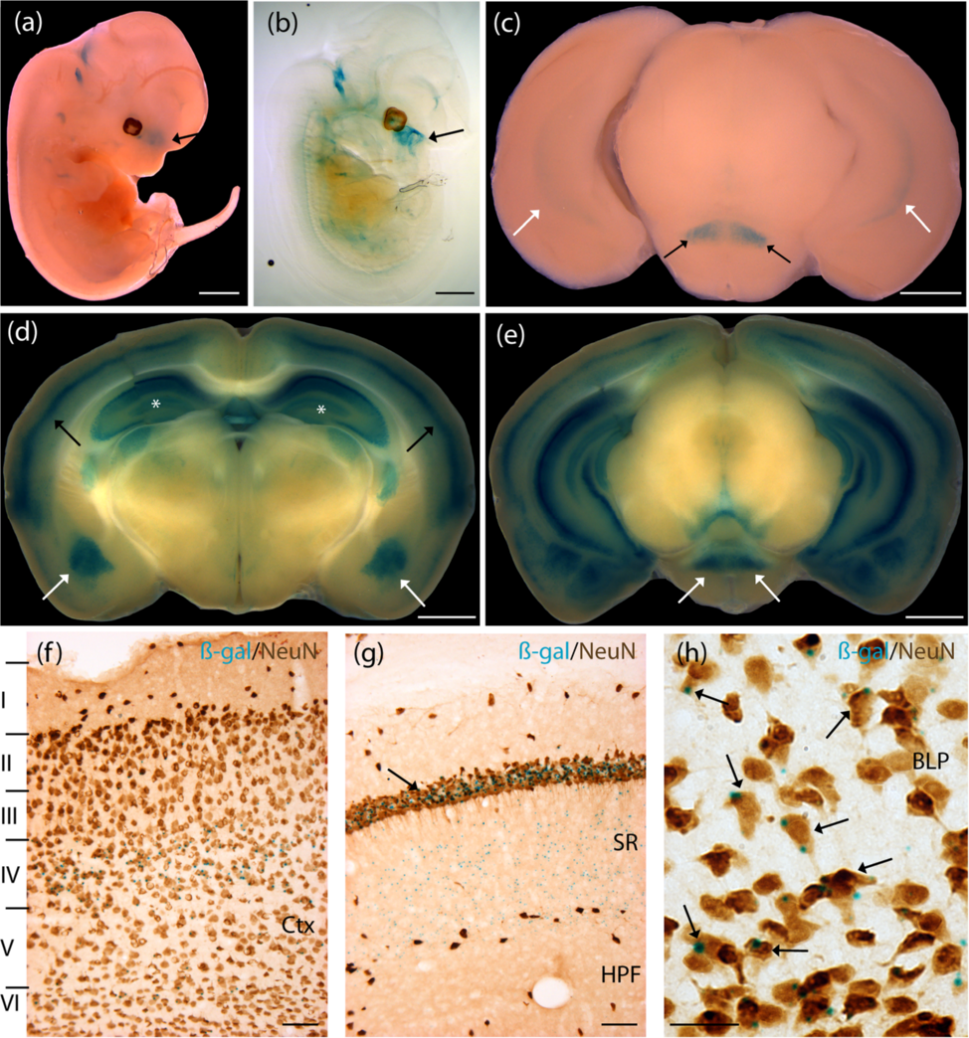
**Human *NOV*-*lacZ *expressed in the basolateral amygdaloid nuclei, mid-cortical layers, and pyramidal neurons of the hippocampal formation.** Expression analysis of the human *NOV*-*lacZ* strain was undertaken by examination of β-galactosidase (β-gal) staining (blue). **(a**,**b)** E12.5 whole and cleared embryos showed staining in the olfactory epithelium, and the primitive nasopharynx (black arrows). **(c)** P7 brains stained in the developing retromammillary nucleus (RM) (black arrows), and hippocampal formation (HPF) (white arrows). **(d)** Adult brains stained in the mid-cortical layers (black arrows), and the HPF (white asterisks). Staining was detected in the basolateral amygdaloid nuclei (BLP) (white arrows). **(e)***NOV*-*lacZ* staining was also present in the adult RM (white arrows). **(f)** Colocalization experiment using β-gal staining and a neuronal nuclei (NeuN) antibody (brown) performed on adult brain cryosections revealed expression of *NOV*-*lacZ* in mature neurons in the cortical layers II, IV and VI. **(g)** Colocalization experiment revealed expression of *NOV*-*lacZ* in pyramidal neurons populating the HPF (NeuN, brown). Staining was found in the cell body in the cornu ammonis 1 region (CA1) (black arrow) and extended in the projections found in the stratum radiatum (SR). **(h)** Colocalization experiment revealed expression of *NOV-lacZ* in mature neurons (NeuN, brown) found in the BLP. Ctx, cortex. Scale bar: **(a**-**e)** 1 mm; **(f**,**g)** 50 μm; **(h)** 25 μm.

*NOV* expression was predicted to be observed in the amygdala and basolateral complex in the adult brain using our approach. Whole mount *lacZ* E12.5 stained and cleared embryos revealed expression throughout the olfactory epithelium and the primitive nasopharynx (Figure 4a,b). P7 developing mouse brain sections demonstrated strong expression of the *NOV* MaxiPromoter reporter construct in the retromammillary nucleus (RM) and light expression in the HPF (Figure 4c). Adult mouse brain sections had high expression levels in the mid-cortical layers, and the HPF (Figure 4d). Pronounced staining was detected in the posterior basolateral amygdaloid nuclei (BLP), and the adult RM (Figure 4d,e). We investigated the nature of the positive cells expressed in the upper cortical layers, HPF, and BLP using colocalization experiments on cryosections. Positive colocalization between the β-gal staining product (blue) and NeuN (brown) revealed that the *NOV* reporter construct was expressed in mature neurons in all brain regions, including the cortical layers II, IV and VI, as well as the HPF, and BLP (Figure 4f-h). The staining pattern in the HPF suggested expression in pyramidal neurons, with staining of the cell body in the CA1 that extended into the apical dendrites found in the stratum radiatum (Figure 4g). A similar experiment, using GFAP as a marker of astrocytes, revealed absence of colocalization of the *NOV*-*lacZ* reporter construct in astrocytes populating the cortex, HPF, and BLP (data not shown).

### Human *NR2F2-lacZ* revealed staining in mature neurons, immunoreactive for the Nr2f2 mouse protein in the basolateral, and corticolateral amygdaloid nuclei in adult brain

**Figure 5:**
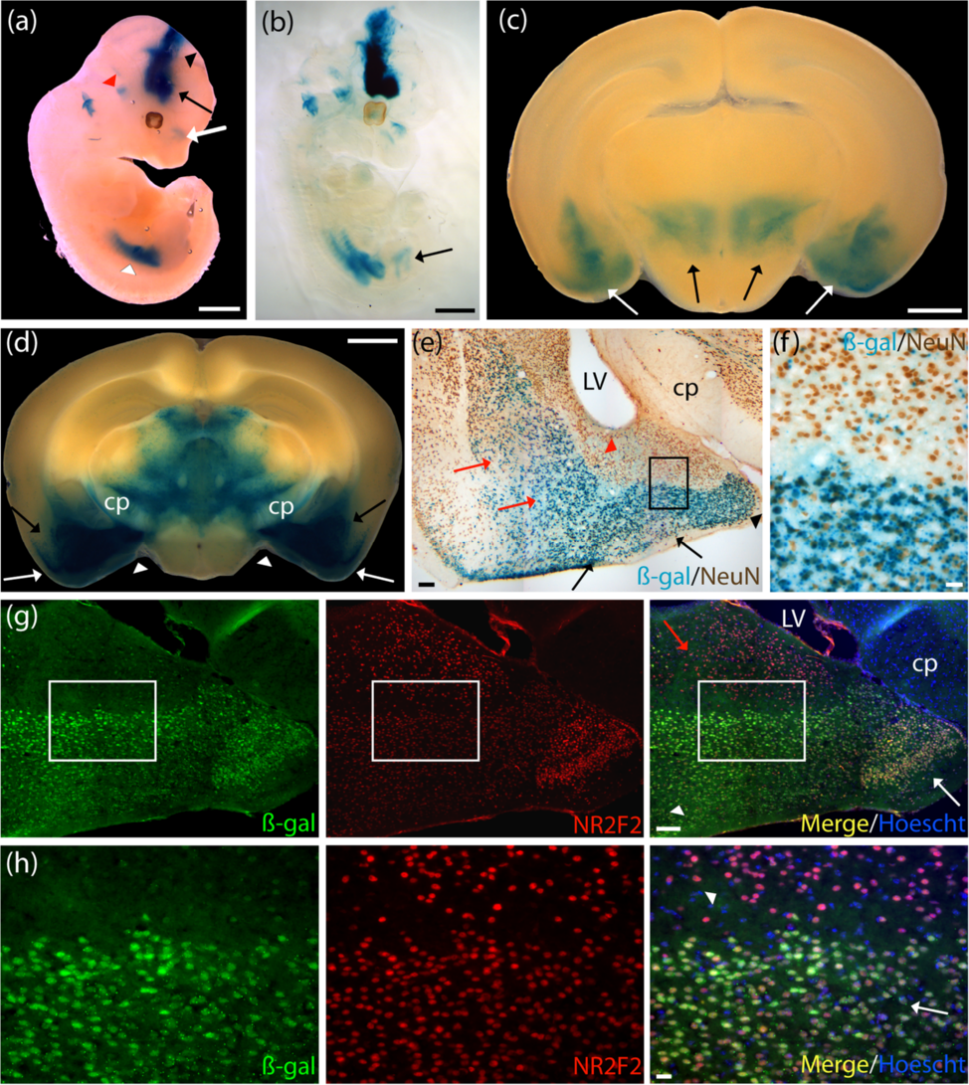
**Human *NR2F2*-*lacZ *expressed in mature neurons populating the basolateral and corticolateral amygdaloid nuclei that are immunoreactive for the Nr2f2 mouse protein.** Expression analysis of the human *NR2F2*-*lacZ* strain was undertaken by examination of β-galactosidase (β-gal) staining (blue). **(a)** E12.5 whole embryos revealed staining in the rostral secondary prosencephalon (black arrow) that extended throughout all three prosomeric regions of the diencephalon (black arrowhead). Staining was present in the nasal cavity (white arrow), the vestibulochochlear ganglion (red arrowhead) and mesenchyme of the posterior limbs (white arrowhead). **(b)** E12.5 cleared embryos additionally demonstrated staining in the developing bladder (black arrow). **(c)** P7 brains stained in the amygdala nuclei (white arrows), and the subthalamic nuclei (black arrows). **(d)** Adult brains revealed strong staining extending from the posterior basolateral amygdaloid nuclei (BLP) (black arrows) to the posterolateral cortical amygdaloid nuclei (PLCo) (white arrows), and the posteroventral part of the medial amygdaloid nuclei (MePV) (white arrowheads). Broad staining was detected in the ventral thalamic area, excluding the cerebral peduncle (cp). **(e)** Colocalization experiment using β-gal staining and a neuronal nuclei (NeuN) antibody (brown) performed on adult brain cryosections revealed strong expression of *NR2F2*-*lacZ* in mature neurons populating the BLP, and the basomedial amygdaloid nuclei (BMP) (red arrows). Colocalization was found in the PLCo, and the posteromedial cortical amygdaloid nuclei (PMCo) (black arrows), and the MePV (black arrowhead). Lower level of β-gal staining was detected in mature neurons in the anterolateral amygdalohippocampal area (AHiAL) (red arrowhead). Boxed region in **(e)** is shown in **(f)**. **(f)** Higher magnification revealed strong expression of β-gal in mature neurons in the PMCo and sparse expression in mature neurons in the AHiAL. **(g)** Colocalization experiment, using an anti-β-gal antibody (green), and an NR2F2 antibody (red), performed on adult brain cryosections revealed strong β-gal labeling in cells expressing the *Nr2f2* mouse gene in brain regions extending from the PMCo (white arrowhead) to the MePV (white arrow). Lower levels of β-gal were detected in the AHiAL (red arrow). Boxed region in **(g)** is shown in **(h)**. **(h)** Higher magnification revealed strong expression of β-gal (green) in *Nr2f2*-positive cells (red) in the PMCo (white arrow) and lower expression in the AHiAL (white arrowhead). LV, lateral ventricle. Scale bar: **(a**-**d)** 1 mm; **(e**,**g)** 100 μm; **(f**,**h)** 20 μm.

*NR2F2* expression was predicted to be in the amygdala and basolateral complex in the adult brain. Whole mount *lacZ* E12.5 stained embryos revealed strong expression of the *NR2F2* MaxiPromoter construct in the rostral secondary prosencephalon that extended throughout all three prosomeric regions of the diencephalon (Figure 5a). Staining was present in the nasal cavity, the vestibulochochlear ganglion, and mesenchyme of the posterior limbs (Figure 5a). E12.5 cleared embryos confirmed staining in the previously described regions, and suggested expression of the *NR2F2* human gene in the developing bladder (Figure 5b). P7 developing mouse brains sections revealed expression of the *NR2F2* reporter construct in the amygdala and subthalamic nuclei (Figure 5c). Adult mouse brain sections exhibited expression in brain regions including the posterior BLP, the basomedial amygdaloid nuclei (BMP), the posterolateral cortical amygdaloid nuclei (PLCo), the posteromedian cortical amygdaloid nuclei (PMCo), and the posteroventral part of the medial amygdaloid nuclei (MePV) (Figure 5d). Strong staining was detected in various thalamic nuclei (Figure 5d). Colocalization experiments on cryosections revealed that the *NR2F2*-*lacZ* reporter gene (blue) was expressed in mature neurons, positive for NeuN (brown), in all brain regions, including the BLP, and BMP (Figure 5e). Colocalization was found in the PLCo, PMCo, and MePV (Figure 5e). Lower levels of *NR2F2*-*lacZ* expression were detected in mature neurons in the anterolateral amygdalohippocampal area (AHiAL), and posteromedial amygdalohippocampal area (AHiPM) (Figure 5e; higher magnification, Figure 5f). The β-gal-positive cells (blue) did not colocalize with GFAP (brown), a marker of astrocytes (data not shown). An additional colocalization experiment, also performed on adult brain cryosections, revealed strong β-gal labeling in cells expressing the *Nr2f2* mouse gene in brain regions extending from the PMCo to the MePV (Figure 5g,h). Lower levels of β-gal were detected in the AHiAL (Figure 5g,h).

### Human regulatory regions specifying expression in adult brain regions of therapeutic interest are functionally conserved from human to mouse

Initially, in choosing the BACs for each gene in this study, and again to understand the relevance of the expression pattern results obtained from the four humanized mouse models, we examined the primary literature and public genomic databases. Specifically, we performed comparative sequence alignment of the BACs against multiple genomes using the University of California, Santa Cruz (UCSC) genome browser [48]. Further, we compared our expression results against the databases of the ABA, BGEM, and *EGFP-*reporter mouse models generated throughout GENSAT.

**Figure 6:**
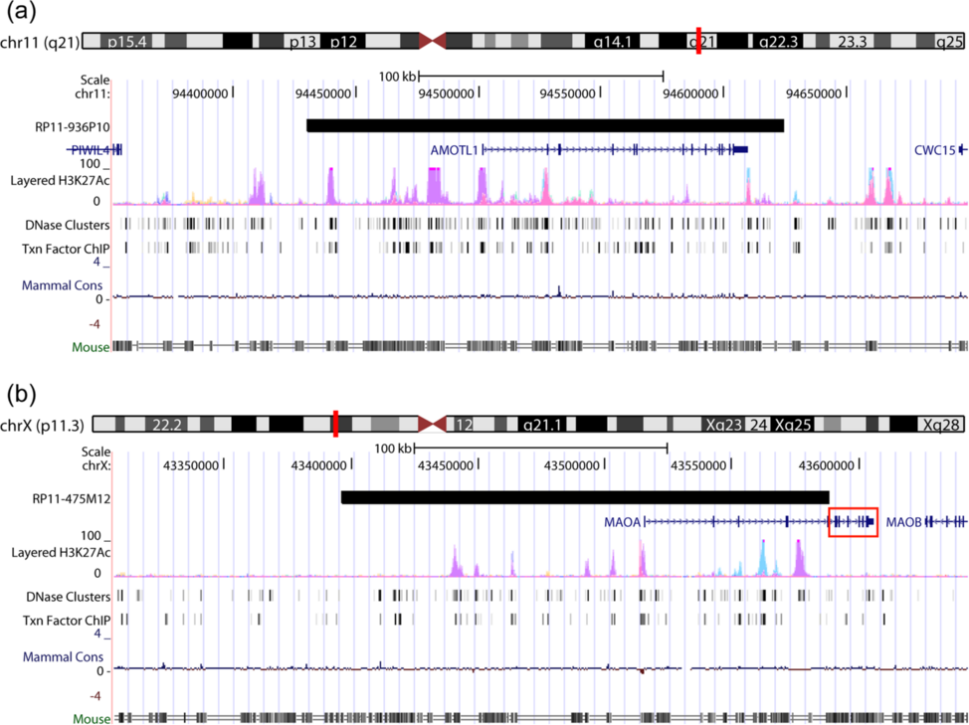
**Comparative genomics delineated the DNA boundaries that were sufficient for adult brain-specific expression of *AMOTL1 *and *MAOA*.** Coordinates corresponding to the human bacterial artificial chromosome (BAC) constructs used in this study were retrieved and visualized using the University of California Santa Cruz (UCSC) genome browser. **(a)** DNA alignment of the human *AMOTL1* BAC (RP11-936P10) with the mouse genome delineated the genomic DNA boundaries sufficient for proper expression of this human gene in the anterior thalamic nuclei in both developing (P7) and adult mouse brain, regions for which this gene was chosen. **(b)** DNA alignment of the human *MAOA* BAC (RP11-475M12) with the mouse genome delineated the genomic DNA boundaries sufficient for proper expression of this human gene in the locus coeruleus (LC) in both developing (P7) and adult mouse brain, regions for which this gene was chosen. One hypothesis suggested by our results was that additional conserved regulatory elements in the 3′ coding and non-coding regions (red rectangle) could be important in narrowing the brain expression of this human gene.

The *AMOTL1*-*lacZ* animals showed staining in the thalamic nuclei at P7, which correlated with available expression data from the ABA at similar developing timepoints (P4 and P14). In contrast, the ABA data showed an absence of expression in the developing HPF for both timepoints suggesting a discrepancy with expression results from our human construct. However, the expression results obtained from the ABA in the adult brain for the *Amotl1* gene did correlate with the results obtained from our human construct, with expression in the thalamic nuclei, and HPF regions CA1 and CA3. Thus, the simplest explanation of the developmental discrepancy in expression in the HPF is a sensitivity difference, with the MaxiPromoter driven *lacZ* being detected earlier than the ABA *in situ* hybridization for endogenous mouse transcripts. This would presume that both are being expressed, but that *lacZ* detection is more sensitive than *in situ* hybridization for the endogenous gene. Since, both studies were performed on the C57BL/6J background; strain differences are unlikely to be a factor. However, it is also formally possible that human regulatory regions important for silencing expression of the *AMOTL1* human gene in the developing HPF are non-functional in mouse, or that they are missing from the MaxiPromoter construct (Figure 6a). Regardless, the key regulatory regions allowing expression of the *AMOTL1* gene in the anteromedial, and anteroventral thalamic nuclei, for which this gene was chosen, as well as the HPF regions CA1 and CA3 in the adult brain, are functionally conserved from human to mouse and were included in our construct.

The *MAOA*-*lacZ* animals showed a staining pattern suggesting expression in the developing LC at P7, which correlated with available expression data from the ABA (P4 and P14) and BGEM (P7) at similar developing timepoints. Staining of adult brain sections in our mouse model revealed expression of *MAOA* in the LC; results which again correlated with the ABA. Colocalization experiments revealed that the *MAOA*-*lacZ* reporter gene was expressed in TH-positive neurons in the LC, which correlated with previously observed results for the mouse gene [21]. Additional staining was detected in the lateral cerebellar nuclei as well as the medial and lateral vestibular nuclei. A careful review of the results obtained by *in situ* hybridization from the ABA revealed low level of expression in the lateral cerebellar nuclei, and absence of expression in the medial and lateral vestibular nuclei. Again, the discrepancies between our results and the ones obtained from the ABA could be attributable to sensitivity differences; with the MaxiPromoter-driven *lacZ* being more easily detected than the ABA *in situ* hybridization for endogenous mouse transcripts. Another possible explanation is that human negative regulatory elements within the BAC are non-functional in mouse. However, in this case we note that the BAC used in the current study was chosen to favor the inclusion of predicted regulatory regions 5′ of the gene, but because of the size of *MAOA* and the available BACs, lacked extensive 3′ coding and non-coding sequences (Figure 6b, red rectangular box). Subsequent to obtaining these expression results, comparative genomic investigation using the UCSC genome browser and focused on the 3′ untranslated region (UTR) of human *MAOA* revealed the presence of a microRNA binding site for a human-mouse conserved microRNA (miR-495, chrX:43606034–43606041). MicroRNAs have been implicated in various biological processes, including gene expression regulation, which argues in favor of including the *MAOA* 3′ UTR region in future gene expression studies [49-53]. Nevertheless, the expression results in TH-positive neurons in the LC in the adult brain showed that the regulatory regions allowing expression of the *MAOA* gene in these cells, for which this gene was chosen, are functionally conserved from human to mouse, and were included in our construct (Figure 6b).

**Figure 7:**
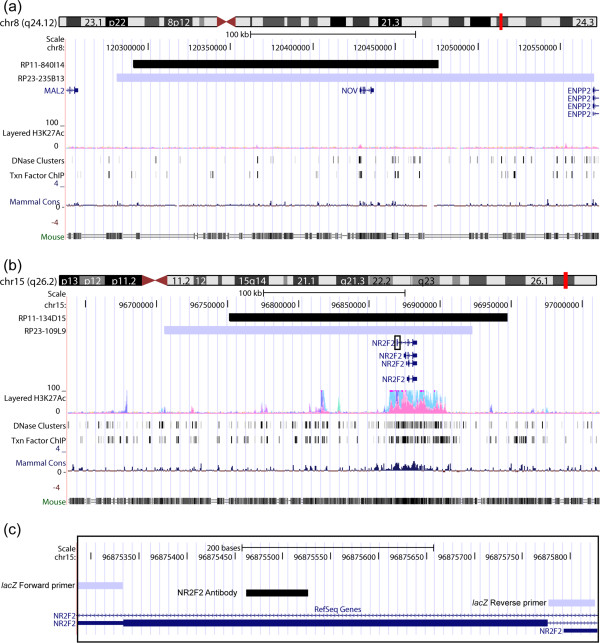
**Comparative genomics delineated the DNA boundaries that were sufficient for adult brain-specific expression of *NOV *and *NR2F2*.** Coordinates corresponding to the human bacterial artificial chromosome (BAC) constructs used in this study were retrieved and visualized using the University of California Santa Cruz (UCSC) genome browser. **(a)** DNA alignment of the human *NOV* BAC (RP11-840I14) (black), against both the RP23-235B13 BAC construct used in the Gene Expression Nervous System Atlas (GENSAT) mouse model (blue), and the mouse genome, delineated the genomic DNA boundaries sufficient for proper expression of this human gene in the basolateral amygdaloid nuclei, cortical layers, and pyramidal neurons in the cornu ammonis 1 (CA1) regions in the adult brain. One hypothesis suggested by our results was that additional functionally conserved regulatory elements homologous to the large non-overlapping 3′ mouse-BAC region are necessary for proper human-gene expression in the developing cortical layers at P7. **(b)** DNA alignment of the human *NR2F2* BAC (RP11-134D15) (black), against both the RP23-109L9 BAC construct used in the GENSAT mouse model (blue) and the mouse genome, delineated the genomic DNA boundaries sufficient for region-specific expression of this human gene in the basolateral, and corticolateral amygdaloid nuclei in the adult brain. One hypothesis suggested by our results was that additional functionally-conserved regulatory elements homologous to the non-overlapping 5′ mouse-BAC region are necessary for proper expression in the developing hypothalamus at P7. Black rectangle box in **(b)** is shown in **(c)**. **(c)** Sequence alignment using the coordinates of the primers used in the BAC *lacZ* retrofitting process (grey bars) and the cDNA sequence used to generate an anti-NR2F2 antibody (black bar), suggested that the absence of expression of the *NR2F2*-*lacZ* constructs in retinal amacrine cells was not attributable to detection of different isoforms of *NR2F2*.

The *NOV*-*lacZ* animals showed staining in the developing RM, and light staining in the HPF at P7, which correlated with available expression data from the ABA (P4 and P14), BGEM (P7), and GENSAT (P7). However, the *NOV*-*lacZ* animals at P7 had no detectable expression in developing cortical layers, which was seen at similar timepoints in ABA, BGEM, and GENSAT. Nevertheless, by adulthood the *NOV*-*lacZ* animals demonstrated strong expression in mid-cortical layers, as well as the HPF, and BLP, results that correlated with those obtained for the ABA, BGEM, and GENSAT datasets. Given the small size of the *NOV* gene, the proximity of neighboring genes, and substantial 5′ and 3′ sequences in the BAC chosen (Figure 7a), the simplest explanation for the temporal delay in expression of our reporter gene in the cortical layers would be a lack of function of human regulatory sequences in mouse, although it is also formally possible that the MaxiPromoter construct is missing regulatory sequences. Building on this latter hypothesis, sequence alignment between the BAC construct used in our study and the one used by GENSAT suggested that the additional regulatory regions may be present in human-DNA homologous to the large non-overlapping 3′ mouse-BAC region. Finally, additional detailed expression analyses revealed staining of the *NOV*-*lacZ* reporter gene in layers II, IV and VI of the cortex, results that correlated with expression data coming from both the ABA, and GENSAT. Expression in the HPF was detected in CA1 pyramidal neurons in our *NOV* mouse model, results that again correlated with the mouse model generated by GENSAT. This suggests that the regulatory regions allowing expression of the *NOV* gene in the adult BLP, the brain region for which this gene was chosen, as well as the cortical layers and HPF, are functionally conserved from human to mouse, and were included in our construct (Figure 7a).

The *NR2F2*-*lacZ* animals showed staining in the developing amygdala, and subthalamic nuclei at P7, which correlated with available expression data from BGEM and GENSAT at the same timepoint. However, *NR2F2*-*lacZ* animals at P7 had no detectable expression in the developing hypothalamus, which was seen at similar timepoints in BGEM, and GENSAT. Nevertheless, by adulthood the *NR2F2*-*lacZ* animals demonstrated expression in the anterior hypothalamic nuclei, as well as the basomedial amygdalar nuclei, results that correlated with those obtained for the ABA, and GENSAT datasets. Again, this temporal delay in expression of our reporter gene in the hypothalamus may be due to lack of function of human regulatory sequences in mouse, or that they are missing from the MaxiPromoters construct.

For *NR2F2*, additional detailed expression analyses included colocalization experiments performed using an antibody for β-gal and an anti-NR2F2 antibody; the latter was raised against the human protein but cross reacted with the mouse ortholog. This analysis revealed specific expression of the *NR2F2*-*lacZ* reporter construct in cells expressing the *Nr2f2* endogenous mouse gene in various amygdala regions. We obtained similar detection levels when comparing the β-gal staining to the endogenous *Nr2f2* mouse gene in regions extending from the PMCo to the MePV in the amygdala. Interestingly, our *NR2F2-lacZ* reporter strain gave lower detection levels in the AHiAL, an amygdala region that showed robust detection levels of the endogenous Nr2f2 mouse protein (Figure 5g,h). This discrepancy could again result from a lack of function of human regulatory sequences in mouse, or the absence of regions that regulate expression level in the AHiAL in our MaxiPromoter construct. Finally, characterization using the NR2F2 antibody revealed Nr2f2 protein in retinal amacrine cells [8]. However, the *NR2F2*-*lacZ* reporter strain demonstrated no expression in the retina (data not shown). In contrast, the GENSAT project reported expression of their *Nr2f2*-*EGFP* reporter strain in retinal amacrine cells (Figure 7b) [54]. Thus, we observed a discrepancy between the results obtained using our reporter mouse model and those from both the endogenous *Nr2f2* mouse gene, and the mouse model generated by GENSAT. One possible explanation was eliminated when sequence alignment of *NR2F2* human gene revealed that the AUG we targeted with our reporter is for the same NR2F2 isoform recognized by the NR2F2 antibody as mapped by previous studies (Figure 7c) [8,10]. Thus, the lack of expression of our construct in retinal amacrine cells is attributable to the lack of function of human regulatory sequences in mouse, or the absence of those regulatory regions in the MaxiPromoter construct. If the latter is the case, we can hypothesize that these regulatory regions may be present in human-DNA regions homologous to the additional 5′ non-overlapping portion of the mouse-BAC construct used in the GENSAT project (Figure 7b). Nevertheless, the expression results in amygdala in the adult brain showed that the regulatory regions allowing expression of the *NR2F2* gene in this region, for which this gene was chosen, are functionally conserved from human to mouse, and were included in our construct.

Despite the minor discrepancies between the expression results of our four different MaxiPromoter constructs and the corresponding expression from the endogenous mouse gene, it is noteworthy that by using bioinformatics predictions, we were successful in each case to delineate the genomic DNA boundaries that were sufficient for expression in the specifically chosen adult brain regions. The delineation of these genomic DNA boundaries is not only important as a proof of principle, but is also crucial for the design of future MiniPromoter constructs for gene-based delivery to these therapeutically important regions.

## Conclusions

Here, we describe an *in vivo* approach by which to further refine our understanding of gene-expression regulation. We first generated a list of ten human genes with expression enriched in brain regions of therapeutic interest, and predicted to have all essential non-coding regulatory regions contained within an identified BAC. We then tested the veracity of these predictions using novel knock-in reporter mouse models. This approach, using the HuGX method, was built on expertise from five specialized laboratories, and scaled to higher-throughput with the help of the pipeline previously designed within the Pleiades Promoter Project [5,7].

For the ten genes chosen, BACs were recovered for nine, and of these BACs eight were fully retrofitted to contain the *Hprt* homology arms and a reporter gene (*lacZ* or *EGFP*). The success rate of 89% (8 out of 9) in the retrofitting steps demonstrates the efficiency of the approach. The success of obtaining correctly targeted ESC clones for all constructs (8/8), at a rate that varied between 20 and 50%, with an average of 35%, was very efficient. The high correct-targeting rate, afforded high selectivity with regard to the ESC clones injected and increased efficiency of obtaining germline transmission, further streamlining the method. Finally, we observed expression from the human gene in mouse for 63% (5/8) genes. Two of the negative results have enabled testable hypotheses to be developed identifying additional critical regulatory regions for those genes.

Four of the five positive mouse strains, including *AMOTL1*-*lacZ*, *MAOA*-*lacZ*, *NOV*-*lacZ*, and *NR2F2*-*lacZ* were characterized in this study. The fifth construct, *NR2E1*-*lacZ*, was the subject of extensive characterization published [45]. For all the positive strains, the expression for the human gene matched the predicted specific adult brain region for which they were chosen. This defined the genomic DNA boundaries that were sufficient for adult brain-specific expression, as well as refined our knowledge regarding the complexity of gene regulation, and demonstrated that a careful investigation, using both elements from publicly available resources and bioinformatics, can lead to accurate prediction of gene expression.

Careful analyses of the expression patterns of these human genes demonstrated slight variations from available mouse expression data. These variations could be a true reflection of species-specific differences in expression. However, they could result from the misuse of human regulatory regions in the mouse environment, or the omission of minor regulatory regions from the BAC construct. Another possible source of difference lies in the effects resulting from the insertion site on the X chromosome. The endogenous *Hprt* locus is widely expressed [40,41], nevertheless, it is considered a relatively neutral docking site since the expected restricted expression is observed from tissue-specific promoters inserted at the locus [5,55-60]. In addition, it has been suggested that the introduction of a larger DNA construct (that is, BAC DNA) would further minimize the risk of influences from the *Hprt* insertion site by providing the essential chromatin environment, thus producing endogenous patterns of gene regulation [61]. Nevertheless, the possibility remains that the slight variation in expression between our knock-in human construct and the mouse gene could be attributable to influence from the *Hprt* locus.

The future of gene therapy may rely upon the development of small human promoters to finely regulate the expression of therapeutic genes in a cell-specific manner. The results from this project delineate, refine, and characterize non-coding-regulatory regions of human genes. Thus, we have characterized the expression pattern, and the non-coding regulatory regions, of four therapeutically important human brain genes. In the near future, refined mouse models using subsets of the regulatory regions defined within these boundaries could lead to the generation of MiniPromoters driving the expression of a gene therapy specifically in the thalamus, locus coeruleus, and various amygdala nuclei in the brain.

## Methods

### MaxiPromoter design

The BAC constructs came from the RPCI-11 human male BAC library [62] accessed 4 January 2012 (BAC numbers, see Table 1). Suitable BACs were selected based on coverage of the gene of interest and its upstream sequence. Candidate regulatory regions were predicted based on sequence conservation and experimental data provided in the UCSC genome browser, and a manual review of the scientific literature. Under the criteria applied, the ideal BAC would cover the entire gene up to, but not including, the promoter regions of neighboring genes. If multiple BACs were available, priority was given to the one that included the most upstream sequence. Two 50 bp oligonucleotide recombination arms were designed for the insertion of the reporter gene in the BAC. The left arm targeted immediately upstream of the endogenous ATG. Ideally, the right arm targeted immediately after the end of the same exon. Because of sequence composition challenges for retrofitting, in some cases the initial right arm oligonucleotide designs were altered to target further downstream.

### BAC retrofitting

BACs were modified by two sequential steps of retrofitting using the lambda recombination system [63]. The first retrofitting step allowed the insertion of *Hprt* homologous recombination targeting arms as we have described previously [45]. The second step allowed the insertion of a reporter cassette (*lacZ* or *EGFP*) at the ATG of the specified gene. Only the *lacZ* or *EGFP* coding sequence, including a STOP codon, was added at the ATG. No intron or polyA signal was added, as the endogenous splicing and polyA were to be used to best reflect the natural gene expression. The specific primers used for the retrofitting of each construct are listed in Table 4. The retrofitting of the reporter cassette was performed as we have described previously [45]. Briefly, the reporter cassette was designed to contain a kanamycin gene, allowing the selection of correctly retrofitted clones. This resistance gene was designed with flanking full *frt* sites [64], which were used to excise the kanamycin gene via induction of FLPe recombinase [65,66]. All newly created DNA junctions, whether for *Hprt* arms or the reporter cassette, were sequence verified. The resulting MaxiPromoter constructs contained a reporter gene under the influence of the human regulatory regions of the specified gene (for a complete list of genes, see Table 1).

**Table 4:** Primers used for reporter-gene retrofitting

**Gene**	**Primers (5′-3′)**	**Sequence**^**a**^
*AMOTL1*	Forward	CCGGCAGCCGTCTTCCCCAGCCGAGGGACTGAACTAGCCATGATCGCCTCatggcggatcccgtcgtttt
	Reverse	GTGGGAGACTCGGAGACGCCCTCCCGGCACCTCGAGTGGGGGCTGGTTACgaagttcctatactttctag
*LCT*	Forward	TTGCAGTTATAAAGTAAGGGTTCCACATACCTCCTAACAGTTCCTAGAAAatggtgagcaagggcgagga
	Reverse	TGTGTGATGAAGGTTGCCGAGGGGTCACCATCAGGTCAATGTGTACTCACgaagttcctatactttctag
*MAOA*	Forward	TTGCCGTCCCCACTCCTGTGCCTACGACCCAGGAGCGTGTCAGCCAAAGCatggcggatcccgtcgtttt
	Reverse	ACCCCTCACTGGCCAGGGTCCCCCAGGCCACCGCTACGGTCCACACTGACgaagttcctatactttctag
*NGFR*	Forward	CCGCAAAGCGGACCGAGCTGGAAGTCGAGCGCTGCCGCGGGAGGCGGGCGatggcggatcccgtcgtttt
	Reverse	GGAGTTCTGATCCCGGGAAAGGGAGCGGGCCCCCTCCGGCTAACACTCACgaagttcctatactttctag
*NOV*	Forward	TACAGCGAAGAAAGTCTCGTTTGGTAAAAGCGAGAGGGGAAAGCCTGAGCatggcggatcccgtcgtttt
	Reverse	ACCAAGGCGGGCAAAGTAACTTGGGGGCATCTTAAGGGTGTGCCACTTACgaagttcctatactttctag
*NR2E1*	Forward	GCCGGGACTCGGGCAGCGCCCACCAACCGCTCCGCCCCGGGACAGCCAGCatggcggatcccgtcgtttt
	Reverse	TCGCCCCAGGCTGCGCGCCTAGGCCCCACGGCGGCCCGAGAGGTACCCACgaagttcctatactttctag
*NR2F2*	Forward	CGCCGCCCGCAGCCAGGGGAGCAGGAAGTCCGGACGCAGCCCCCATAGATatggcggatcccgtcgtttt
	Reverse	CCAGGACCCCGGGACCCAGGACGAGGGAAGGAGAAATGAGAGGCCGATACgaagttcctatactttctag
*PITX2*	Forward	CCGCCGCTTCTTACAGCCTTCCTTCTCTTCTGTTTTGCAGATAACGGGGAatggcggatcccgtcgtttt
	Reverse	GTGGCGCGGCCTCCCGTCCGATGACCCGGGCAGGAGAAGGGGGTTCTTACgaagttcctatactttctag

^a^Capital letters, sequence matching the mouse genome; lowercase letters, sequence matching the reporter gene (forward primer) or the kanamycin cassette (reverse primer).

### Mouse strain generation, husbandry, and breeding

The strains were generated using a variation of the previously described strategy to insert constructs 5′ of *Hprt* on the mouse X chromosome (HuGX) [7,46,61,67]. Briefly, BAC DNA was purified using the Nucleobond BAC 100 kit (Clontech Laboratories, Mountain View, CA, USA) and linearized with *I-Sce*I. The BAC constructs were electroporated into ESCs using the following conditions: voltage, 190 V; capacitance, 500 μF; resistance, none; using a BTX ECM 630 Electro cell manipulator (BTX, San Diego, CA, USA) [45]. ESC clones were selected in hypoxanthine aminopterin thymidine (HAT) media, isolated, and DNA purified. Human-specific PCR assays were designed to be on average 8 kb apart (range 0.334 to 20) throughout the BAC construct, and used to screen the ESC clones as well as verify the integrity of the BAC inserted into the mouse genome [45,46]. Table 2 lists all the ESC lines used and their associated genotype. ESC derivation and culture was conducted as we have described previously [46]. Correctly targeted ESC clones were microinjected into B6(Cg)-*Tyr*^*c-2J*^/J (B6-Alb) (JAX Stock#000058) blastocysts to generate chimeras that were subsequently bred to B6-Alb females to obtain female germline offspring carrying the BAC insert. The female germline offspring were then bred to C57BL/6J (B6) (JAX Stock#000664) males and backcrossing to B6 continued such that mice used in this study were N3 or higher. Table 3 list the details about the strains used in this study, these are available at The Mutant Mouse Regional Resource Center (MMRRC) [68]. Male animals were used in all studies to avoid any variability due to random X-inactivation of the knock-in alleles at *Hprt*, and the age of the adult animals used in this study ranged from P51 to P305.

For the cre/loxP experiment, the ACTB-cre allele was obtained from strain FVB/N-Tg(ACTB-cre)2Mrt/J (JAX Stock#003376) and then backcrossed to B6 such that mice used in the study were N10 or higher. Females, heterozygous for the human BAC MaxiPromoter reporter genes (*Hprt*^*tm66(Ple5-lacZ)*Ems/+^, *Hprt*^*tm68(Ple127-lacZ)Ems*/+^, *Hprt*^*tm69(Ple134-lacZ)Ems*/+^, *Hprt*^*tm75(Ple143-lacZ)Ems*/+^) were bred to males heterozygous for the ACTB-cre gene (ACTB-cre/+). The resulting offspring contained experimental males, hemizygous for the reporter genes, and heterozygous for the ACTB-cre gene (*Hprt*^*tm66(Ple5-lacZ)*Ems^/Y, ACTB-cre/+; *Hprt*^*tm68(Ple127-lacZ)Ems*^/Y, ACTB-cre/+; *Hprt*^*tm69(Ple134-lacZ)Ems*^/Y, ACTB-cre/+; *Hprt*^*tm75(Ple143-lacZ)Ems*^/Y, ACTB-cre/+), and control males, hemizygous for the MaxiPromoter reporter genes only (*Hprt*^*tm66(Ple5-lacZ)*Ems^/Y, +/+; *Hprt*^*tm68(Ple127-lacZ)Ems*^/Y, +/+; *Hprt*^*tm69(Ple134-lacZ)Ems*^/Y, +/+; *Hprt*^*tm75(Ple143-lacZ)Ems*^/Y, +/+). Animals of the appropriate genotype were kept and processed for *lacZ* staining as described below.

All mice were maintained in the pathogen-free Centre for Molecular Medicine and Therapeutics animal facility on a 7 AM to 8 PM light cycle, 20 ± 2°C with 50 ± 5% relative humidity, and had food and water *ad libitum*. All procedures involving animals were in accordance with the Canadian Council on Animal Care (CCAC) and UBC Animal Care Committee (ACC) (Protocol# A09-0980 and A09-0981).

### Embryo and adult tissue preparation

Time-pregnant mice were killed by cervical dislocation and embryos at E12.5 were dissected, and then fixed in 4% paraformaldehyde (PFA) with 0.1 M PO (0.1M Na_2_HPO_4_) buffer (pH 7.2 to 7.4) for 4 h at 4°C. Whole embryos were incubated in *lacZ* staining solution (X-gal (1 mg/ml), MgCl_2_ (2 mM), K_3_Fe(CN)_6_ (4 mM), K_4_Fe(CN)_6_ (4 mM) in 1 × phosphate-buffered saline (PBS)) overnight at 37°C and were subsequently washed in three volumes of 1 × PBS before being photographed. Embryos having the desired genotype were cleared as described in the literature [69] and pictures were taken in 100% glycerol solution.

Intracardial perfusions were performed on avertin-anesthetized mice with 4% PFA with 0.1 M PO buffer (pH 7.2 to 7.4). Brain tissues destined to be 1 mm sectioned were collected and post fixed in 4% PFA for an additional 2 h at 4°C before being sectioned in a rodent brain matrix (RBM-2000S/RBM-2000C, ASI Instruments, Michigan, USA). The sectioned brains were incubated in *lacZ* staining solution for a duration varying between 2 h to overnight at 37°C and were washed in 1 × PBS before being photographed. Brain tissues destined to be cryosectioned were directly transferred to 20% sucrose with 0.05 M PO buffer overnight at 4°C and embedded the next day in optimal-cutting-temperature (OCT) compound (Tissue-tek, Torrance, CA, USA) on dry ice. Eye tissues destined to be cryosectioned were incubated in *lacZ* staining solution overnight at 37°C and were washed in 1 × PBS before being post fixed for 2 h in 4% PFA at 4°C. The eyes were washed in 1 × PBS, then transferred to 20% sucrose with 0.05 M PO buffer overnight at 4°C and embedded the next day in OCT on dry ice.

### Histology

Brain structures were identified using *The Mouse Brain in Stereotaxic Coordinates*, third edition [70].

For immunofluorescence, 25 μm cryosections from adult brains (floating sections) were rehydrated in sequential washes of PBS, permeabilized in PBS with 0.1% triton, and quenched in 0.1 M glycine-PBS solution. The cryosections were blocked with 1% BSA in PBS with 0.1% triton for 1 h at room temperature before applying the primary antibodies. Colocalization experiments were performed using chicken anti-β-gal antibody (Abcam, San Francisco, CA, USA; ab9361) 1:5,000, mouse anti-NR2F2 antibody (R&D systems, Minneapolis, MN, USA; PP-H7147-00) 1:100, and incubated overnight at 4°C. Corresponding secondary antibodies coupled to Alexa 488 or Alexa 594 (Invitrogen, Burlington, Ontario, Canada) were incubated at room temperature for 2 h in the dark (1:1,000). Hoechst 33342 was used for nuclear staining on all immunofluorescence sections.

For immunohistochemistry staining of adult brain, 25 μm cryosections (floating sections) were rehydrated in sequential washes of PBS, permeabilized in PBS with 0.1% triton before being incubated in *lacZ* solution overnight at 37°C. The following day, the sections were rinsed, post fixed in 2% PFA for 10 minutes, and blocked with 0.3% bovine serum albumin (BSA), 10% normal goat serum solution for 20 minutes. Primary antibodies were incubated overnight at 4°C using the following dilutions: rabbit anti-TH antibody (Pel-freez Biologicals, Rogers, AR, USA; P40101-0) (1:500), mouse anti-NeuN (Millipore, Billerica, MA, USA; MAB377) (1:500), mouse anti-GFAP (New England BioLabs, Cell Signaling Technology, Danvers, MA, USA; mAB3670) (1:200), rabbit anti-Brn3 (recognizing Brn3a, Brn3b, Brn3c, also known as Pou4f1, Pou4f2, Pou4f3) (Santa Cruz, Dallas, TX, USA; sc-28595) (1:1,000), rabbit anti-calbindin (Abcam, San Francisco, CA, USA; ab49899) (1:1,000). The third day, sections were rinsed and corresponding secondary antibody coupled to biotin (Vector Laboratories, Burlingame, CA, USA) were incubated at room temperature for 1 h (1:200). The sections were finally processed for standard avidin-biotin immunocytochemical reactions using the ABC kit from Vector Laboratories. Immunolabeling was visualized using 3.3-diaminobenzidine tetrahydrochloride (DAB) (Roche, St Louis, MO, USA). Sections were dehydrated in subsequent washes of 50%, 70%, 95%, 100% ethanol, and xylene before being mounted for microscopy.

For adult eyes, stained with *lacZ*, 20 μm cryosections were mounted directly on slides and pressed for 30 minutes before being processed for counterstaining or antibody labeling. Antibody staining was performed as previously described for floating brain sections. Counterstaining was performed using neutral red as follows: slides were washed once in PBS for 2 minutes, followed by a 2-minute wash in water. They were then incubated for 45 s in neutral red solution before being subsequently dehydrated in 70% to 100% ethanol and xylene and then mounted for microscopy.

## Abbreviations

5-HT: 5-hydroxytryptamine (serotonin); ABA: Allen mouse brain atlas; ACC: Animal care committee; AHiAL: Anterolateral amygdalohippocampal area; AHiPM: Posteromedial amygdalohippocampal area; AMOTL1: Angiomotin-like 1; BAC: Bacterial artificial chromosome; BGEM: Brain gene expression map; BLP: Basolateral amygdaloid nuclei; BMP: Basomedial amygdaloid nuclei; BSA: Bovine serum albumin; CA1: Cornu ammonis 1; CA3: Cornu ammonis 3; CCAC: Canadian Council on Animal Care; CGE: Caudal ganglionic eminence; CNS: Central nervous system; DAB: 3.3-diaminobenzidine tetrahydrochloride; ENCODE: Encyclopedia of DNA Elements; ESC: Embryonic stem cell; GCL: Ganglion cell layer; GENSAT: Gene expression servous system atlas; HAT: Hypoxanthine aminopterin thymidine; HD: Huntington’s disease; HPF: Hippocampal formation; HuGX: High-throughput human genes on the X chromosome; ILM: Inner limiting membrane; INL: Inner nuclear layer; IPL: Inner plexiform layer; JEAP: Junction-enriched and -associated protein; LC: Locus coeruleus; MAOA: Monoamine oxidase A; MBP: Medial parabrachial nucleus; MePV: Medial amygdaloid nuclei; MMRRC: Mutant mouse regional resource center; NOV: Nephroblastoma overexpressed gene; NR2F2: Nuclear receptor 2F2; OLM: Outer limiting membrane; OPL: Outer plexiform layer; PBS: Phosphate-buffered saline; PFA: Paraformaldehyde; PLCo: Posterolateral cortical amygdaloid nuclei; PMCo: Posteromedian cortical amygdaloid nuclei; RM: Retromammillary nucleus; SR: Stratum radiatum; TH: Tyrosine hydroxylase; TSS: Transcription start site; UCSC: University of California Santa Cruz.

## Competing interests

The authors declare that they have no competing interests.

## Authors’ contributions

SJMJ, WWW, DG, RAH, and EMS conceived and initiated the project. EPC and WWW performed bioinformatics analysis to select BACs. EPC designed the BAC retrofitting procedure. MC and GW generated the modified BAC constructs. SL, KGB, RJB, MA and AJK generated the mice and maintained the colonies. JFS, SL, KGB, RJB performed adult tissue collections and *lacZ* staining. JFS, SCM and LB performed adult tissue cryosectioning, *lac*Z staining, and immunohistochemistry. JFS and KGB performed embryo collection, *lacZ* staining and clearing. JFS performed the expression pattern characterization in embryos and adult tissues. JFS performed all data analyses, wrote the text, and created all of the figures for this manuscript. MC, SL, KGB, RJB, EPC, WWW, DG, RAH, and EMS revised the manuscript prior to submission. All authors read and approved the final manuscript.
